# Sildenafil in Infants and Children

**DOI:** 10.3390/children4070060

**Published:** 2017-07-24

**Authors:** Larisa Simonca, Robert Tulloh

**Affiliations:** Department of Congenital Heart Disease, Bristol Royal Hospital for Children, Bristol BS2 8BJ, UK; simonca.larisa12@gmail.com

**Keywords:** pulmonary hypertension, Sildenafil, phosphodiesterase inhibitor

## Abstract

Pulmonary arterial hypertension (PAH) management has been transformed in recent times with the advent of cheap and effective diagnostic tools and therapy. Sildenafil, a phosphodiesterase-V inhibitor, has been at the centre of this treatment, and its success in treating PAH has led to its widespread uptake in adult and paediatric pulmonary hypertension (PH), as a first line treatment choice. This might apply to persistent pulmonary hypertension of the newborn (PPHN) or bronchopulmonary dysplasia, as well as to more complex diseases, such as idiopathic pulmonary hypertension. Although recent data regarding long-term mortality and the repeal of Food and Drug Administration (FDA) approval has complicated the issue, Sildenafil continues to be the major treatment option for paediatric PH for patients in a variety of contexts, and this does not seem likely to change in the foreseeable future. In this review, we provide a summary of pulmonary hypertension in infants and children and the use of Sildenafil for such diseases.

## 1. Introduction

Pulmonary hypertension (PH) is a chronic pathology with a progressive nature that, without treatment, can cause various complications, such as right-sided heart failure and death [1]. Often screened by means of echocardiography, a mean pulmonary arterial pressure over 25 mm Hg at rest, observed at the catheterization of the right heart, defines this condition [2]. Any child who experiences shortness of breath, who tires easily, or has syncopal episodes, should be suspected of suffering from PH, even in the presence of heart or lung disease. Making the correct diagnosis is important in order to effectively treat and improve the quality of life for such patients. An echocardiogram that shows a tricuspid regurgitation at a peak Doppler velocity exceeding 2.7 m/s [3], a chest radiograph that reveals reduced peripheral pulmonary vascularity, as well as increased central pulmonary arteries and electrocardiographic markers of right ventricular dilatation or hypertrophy, are the essential findings used to suggest PH [2].

There are a large number of aetiologies that can lead to PH, and although nowadays the majority of the risk factors are known, there are still an important number of cases in which the cause has yet to be discovered. In all cases, PH is a result of the increased resistance to blood flow through the lungs, which is sometimes due to the increased volume of pulmonary blood flow. Therefore, any change in either or all of these variables will consequently cause an increase in pulmonary arterial pressure (Table 1) [4].

Five groups of conditions have been classified as leading to PH: (1) pulmonary arterial hypertension (PAH); (2) a reaction to left-sided heart disease; (3) illnesses that emerge from either lung diseases or hypoxia; (4) chronic thromboembolism and (5) a grouping for unclear, unknown, multifactorial mechanisms [5].

Because of the severity of PH and its complications, it is suggested that treatment is initiated as soon as possible. Without treatment, the natural evolution of the condition leads to right-sided heart dysfunction, failure and death [6]. Unfortunately, there is currently only limited data on therapeutic options for PH. However, recent studies have now shown promising results for the use of phosphodiesterase type 5 inhibitors (PDE5 inhibitors), of which Sildenafil has emerged as a top potential therapy.

## 2. Sildenafil Overview

Sildenafil, a PDE5 inhibitor with high selectivity, increases the level of cyclic guanosine monophosphate (cGMP) in the body [7,8,9]. The accumulation of cGMP then leads to a series of cellular changes that concludes with a decrease in intracellular calcium levels and the relaxation of smooth muscles [10,11]. New studies suggest that Sildenafil also improves right ventricular function, which reduces vascular smooth muscle contraction (VSMC), as well as proliferation by modulating the Rho-associated kinase signalling [12,13,14]. PDE5 is found in various locations in the body: corpus cavernosum, retina, platelets, smooth muscles of the vascular system and in high levels in the pulmonary circulation [9,15]. 

Sildenafil was first approved for erectile dysfunction in 1998, but since then, additional uses for this drug have also been found. In 2005, it was approved for PH in adults and consequently an interest in research arose, questioning whether or not it could also be used successfully in infants and children [16]. Today, Sildenafil is considered an encouraging treatment in PH in infants and children, although it is still used off-label with the need for further studies. Therefore, it should only be administrated under careful observation of a paediatric cardiologist [17].

### 2.1. Pharmacokinetics and Pharmacodynamics 

The pharmacokinetics of Sildenafil continue to be of interest in different clinical situations and new ways of administration are being pursued. Most of the information that is available today applies to oral administration. However, sublingual administration, in which the first-pass metabolism was partially bypassed, could be a useful alternative, due to the fact that plasma concentration is increased by over 300%, showing that this route of administration has better outcomes compared to enteral administration [18]. Additionally, sublingual administration can cause a notable increase in effective oxygenation compared to intravenous infusion, although further trials are still required [19]. When parenteral administration is necessary due to various conditions, and intravenous (IV) Sildenafil is not available, administration of a solution consisting of Sildenafil and a facility-approved fluid can be considered via a nasogastric tube [20].

### 2.2. Absorption

The quick absorption, between 0.5–1.5 h, together with the bioavailability for oral administration of Sildenafil (38–41% in adults), makes it a great route of administration due to the good compliance that is shown in patients. Additionally, an increased absorption and bioavailability has been noted when Sildenafil is administrated sublingually [18]. Furthermore, oral administration has significantly lower costs [21], since even though bioavailability of intravenous use is 100%, increased costs are encountered using IV administration.

### 2.3. Metabolism

Sildenafil is metabolized in the liver, through specific enzymes (CYP3A4 and CYP2C9), and UK-103 320 is released in circulation as the main metabolite, acquiring 50% of Sildenafil’s potency. In newborns, another important factor is the CYP3A7 enzyme, whose levels are the highest after birth and decrease simultaneously with the increase of CYP3A4 and CYP2C9 during the first week of life [22]. This developmental change that occurs in the first few weeks of life might explain the large inter-patient variability of the response to Sildenafil, but this has yet to be concluded and requires further investigation. Either route of administration that is being used, whether oral or intravenous, results in a half-life ranging from 2–4 h, placing Sildenafil into the short-acting drug group that reaches the maximum plasma concentration after 0.5–2.5 h, targeting a 50–400 ng/mL level of plasma concentration [16,21,23]. It is known that the clearance of Sildenafil metabolites increases threefold in the first few days of life and the volume of distribution is fourfold greater than in adults. This leads to a much longer terminal half-life in neonates [24]. This effect on clearance of Sildenafil in neonates in their first week of life also shows the importance of CYP enzyme developmental changes that occur with age in healthy subjects [25,26]. In case of liver or renal associated pathologies, studies have shown a significant decrease in clearance [25], showing much longer time for excretion and consequently lower Sildenafil clearance has been observed in PH patients [27]. This suggests that in those patients with reduced renal function, the starting dose should be reduced in order to allow for effective clearance of both sildenafil and its metabolites.

### 2.4. Side Effects and Complications

Pulmonary hypertension has been a difficult condition to treat for a long time, most especially in infants and children, where ethical aspects are exceedingly complex and research studies face many restrictions. Since the approval of Sildenafil in adults, specialists have shown an interest in using the drug in infants and children as well. However, there still remains a constant struggle to find the right balance between risks and benefits in Sildenafil treatment.

Considering Sildenafil’s mechanism of action, which decreases pulmonary arterial pressure, concerns have been raised that Sildenafil could cause serious systemic hypotension and severe haemodynamic instability. Indeed, Sildenafil was first considered as an anti-hypertensive agent. To this day, this effect has not been significantly proven, although there are a few case reports that mention moderate hypotension related to Sildenafil treatment. Additionally, the vasodilatation induced by the blockage of PDE5 inhibitors can lead to nasal congestion, as well as flushing, and can also lead to headaches [3].

Another controversial aspect of Sildenafil is its activity on the PDE6 receptors localized in the rod and cone cells of the eye, and whether or not the drug affects the normal development of the visual function of preterm neonates. However, there has been no proven evidence of a direct link between Sildenafil therapy and ocular complications, although photophobia has been observed in some subjects [3,27,28,29].

Common side effects of Sildenafil treatment are usually non-life-threatening. Upper respiratory tract infections were the most frequent of the adverse reactions to the treatment, followed, in order of frequency, by vomiting, headache, bronchitis, pyrexia, pharyngitis, cough, diarrhoea and nasopharyngitis (nasal stuffiness) (Table 2) [30]. Severe adverse effects that were treatment-related and that required immediate termination of the treatment, only appeared in five subjects of the double-blind, placebo-controlled Sildenafil in Treatment-Naive Children with Pulmonary Arterial Hypertension (STARTS-1) study and consisted of stridor and ventricular arrhythmias.

### 2.5. Indications for Sildenafil in Infants and Children

The guidelines of the European Society of Cardiology (ESC) [1] for PH recommend Sildenafil therapy in children, for those aged 1–17 years old. These recommendations state that once diagnosis is confirmed of Group 1 PH (PAH), those children that fit the criteria for World Health Organisation Functional Class (WHO FC) II and III, could benefit from Sildenafil monotherapy three times a day [1]. The intermediate and high-risk forms, that correspond to the WHO FC III and FC IV, should be treated with combinations of endothelin receptor antagonists (such as Bosentan, Macitentan and Ambrisentan) Sildenafil (or other PDE5 inhibitors) and possibly intravenous epoprostenol. If this treatment fails, double or triple sequential combinations are recommended [1].

## 3. Persistent Pulmonary Hypertension of the Newborn

The use of Sildenafil in Persistent Pulmonary Hypertension of the Newborn (PPHN) is controversial and needs further investigation, mainly due to the high risk of hypotension that was observed in IV administration. Therefore, it is highly recommended that it should be administrated in a slow IV infusion to reduce the occurrence of such hypotension. Sildenafil has been shown to improve arterial oxygenation by 16 mmHg [31] in patients with PPHN, an effect that was observed with or without previous exposure to nitric oxide (NO) [32], which diminishes the risk of mortality. It has also been recognized that weaning of NO during the recovery period is associated with rebound PH, and Sildenafil has been found to ameliorate this rebound that is observed during inhaled nitric oxide (iNO) weaning [33].

### 3.1. Bronchopulmonary Dysplasia in Infants and Children

Bronchopulmonary dysplasia (BPD) is a chronic lung disease, more frequently seen in premature infants. Prematurity is observed in 10% of all births and BPD is seen in approximately 10% of these, of whom PH is found in 30–45% of these cases [34,35]. About half of those children who have BPD-PH will die as a result. A remaining challenge is to find a treatment that will improve the quality of life and the survival of BPD patients, taking into account that even after treatment with the most commonly used vasodilator agent (oxygen), the mortality is still 50% [36]. Even though NO can serve as an effective therapy, treatment is costly and long-term therapy can be difficult, as it is an inhaled toxic gas. 

Some studies have shown that PDE5 inhibitors normalize lung development, decrease right ventricle peak systolic pressure and improve outcomes [37,38]. Sildenafil can be used as a monotherapy in BPD associated with PH, but only with close supervision and in situations that have no other option. Sometimes there is need for a combination with other drugs, such as NO, endothelin-receptor antagonists and Prostanoids [39]. Currently however, there has yet to be found significant evidence from a randomized controlled trial (RCT) that Sildenafil improves long-term morbidity or mortality from BPD-PH.

### 3.2. Congenital Diaphragmatic Hernia in Infants and Children

Pulmonary hypertension associated with congenital diaphragmatic hernia (CDH) in infants and children is a challenging condition to treat. Firstly, the enteral administration of Sildenafil is compromised in the majority of cases, mainly because of the delay in feeding and the food intolerance that can occur [19]. Secondly, the use of Sildenafil in PH associated with CDH is still an off-label use (as are many of the paediatric uses) and does not have enough evidence-based data, therefore it is mandatory to further investigate the treatment effects. The drug could have a potential for therapy for PH associated with CDH, as Bialkowski et al. stated that after 72 h of intravenous Sildenafil use, improvements in oxygenation were observed and that the cardiovascular and respiratory functions benefitted from an early phase treatment [19].

### 3.3. Eisenmenger’s Syndrome in Infants and Children

Eisenmenger’s Syndrome is defined as an untreated septal defect that leads to increased pulmonary vascular resistance (PVR) and subsequently to PAH, due to the reversed or bidirectional blood flow through the defect [40,41]. According to the ESC and UK commissioning guidelines, Sildenafil is recommended as first line treatment in those patients to the patients in WHO FC III [42]. Similarly, sildenafil is regarded as standard therapy in the American Heart guidelines along with caution over the use of excessive dose (see below) [43]. Improvements in haemodynamic parameters and the increase in exercise capacity has been observed when a combination of Sildenafil and Bosentan was used on patients with Eisenmenger Syndrome [44]. Currently, however, there is still little evidence of the role of dual therapy in such patients, the effect of drug-drug interaction and any evidence of long term survival benefit in the paediatric population with the use of long-term PDE5 inhibitor therapy.

### 3.4. Lymphatic Malformations in Infants and Children

Sildenafil in the treatment of lymphatic malformations represents a new therapeutic avenue, although this indication needs further studies. After 20 weeks of therapy, one patient who had obstructive sleep apnoea, airway occlusion and the right ear canal infiltrated by a lymphatic malformation, showed improved symptomatology. Additionally, symptoms improved in 6/7 patients treated for this condition, suggesting a potential significant efficacy. There were no serious adverse effects in any of the patients and none of them had to stop their treatment [45].

## 4. Other Causes of Pulmonary Arterial Hypertension

There have also been a variety of other causes of PAH where children have been treated effectively with Sildenafil. For the patients who experienced high PVR after heart transplant, Sildenafil treatment improved the right ventricular dysfunction and decreased PVR [46]. This is a complex scenario, and relates to the multifactorial cause of PAH in such patients, from the pulmonary complications of transplantation, anti-rejection medication, increased systemic vascular resistance and also reduced ventricular function. Currently, it is not clear exactly how Sildenafil interacts in all of these different areas and additional studies are consequently needed.

Following the Fontan operation, for children and adolescents with functionally univentricular hearts, some patients have decreased pulmonary vascular compliance. In the absence of true PAH, but PVR is increased, Sildenafil or Bosentan therapy administered before and after Fontan surgery, has appeared to benefit such patients. This is now standard therapy both early on in intensive care, but also later in the “failing Fontan” period [47,48].

Five children who underwent haematopoietic stem cell transplant developed PH at a median of 2.5 months post-transplant. One of the children (the only one who survived) received Sildenafil therapy for 11 months, and was off oxygen and doing well after two years from the transplant. Although is an isolated case, this could open the door for Sildenafil use in many more conditions and underlines the importance of further investigations on the subject [49].

## 5. Dosage

Due to the increasing variety of pathologies in which Sildenafil is being used, and to the high number of comorbidities that are found in some of the paediatric patients, a unique dose regimen is almost impossible to establish, often leaving this matter to the judgment of the paediatric cardiologist. However, the approved and recommended dose regimen for oral administration varies according to body weight. In recent studies (STARTS 1 and 2), children were randomized in 4 dose groups of Sildenafil (low, medium, high and placebo) and comparison was made for survival between these groups. In total, a death rate of 16% was found, while the low dose category had the lowest percentage of deaths, at 9%. The medium category was found to have a death rate of 16%, and the high dose group had the highest mortality, as 22% of the patients died, who were treated with a high dose. Interestingly, regardless of the up-titrations that were conducted in the low dose group, the number of deaths in this group was found to be far less than the other groups [16]. 

Therefore, all patients over 1 year and under 20 kg should receive 10 mg of Sildenafil, three times a day. Even though it might be expected that older children should receive a higher dose, there have been concerns from the US that there was an excess mortality in children receiving high dose Sildenafil for PH [50]. As a result, the recommended dosage remains at a maximum administrated low dose, of 10 mg Sildenafil, three times daily. At this dose regimen, children did not show any significant improvements in haemodynamics or in exercise capacity and did not ameliorate their WHO FC, however a lower number of deaths is observed, compared to higher dosages [30].

## 6. Contraindications for Using Sildenafil in Infants and Children

Sildenafil cannot be used in severe cases of hypotension because of its vasodilatory effect. Its use is also contraindicated in left ventricle outflow obstruction, in PAH associated with sickle cell anaemia, as well as in pulmonary veno-occlusive disease. Due to the relative potency of Sildenafil on PDE6, it also cannot be indicated in ischaemic optic neuropathy and in hereditary degenerative retinal disorders [51]. Although it is not the topic of this paper, there are other PDE5i which can be used in situations where there is not a known class effect of contraindications. Tadalafil is now a standard therapy, with the advantage of daily use [1]. There is now good knowledge base for its use in children with few complications [52]. Overall however, the PDE5i have a good track record for their safety and efficacy as a group [53].

## 7. Interaction with Other Drugs Used in the Treatment of Pulmonary Arterial Hypertension

Treatment of PAH in infants and children represents a real challenge and sometimes requires a combination of drugs in order to improve patient outcomes. Regardless of the aetiology of PAH, WHO FC and clinical parameters showed improvement after treating the patients with Sildenafil and Bosentan [54]. However, when treating a patient with more than one drug, interactions may occur and can be a significantly and negatively affect the clinical outcome, rendering the risk of inefficacy or toxicity of the compound. 

Negative interactions have not yet been established when combined therapy of the newer endothelin receptor antagonist Macitentan and Sildenafil was used to treat PAH in adults or paediatric patients [55]. The first breakthrough in treating PAH came with the discovery of Epoprostenol, an intravenously administrated prostacyclin analogue. Both in idiopathic PAH and that associated with congenital heart disease, Epoprostenol showed favourable results, improving survival, as well as reducing their symptoms [56,57,58,59,60]. Although its results are very promising, long-term treatment is possible only in well-funded centres, because of the high cost and also because it can only be administrated with supervision from a specialist. Therefore, in combination with IV treatment with Epoprostenol, Sildenafil offers improved outcome to patients [60].

## 8. Morbidity and Mortality

To this date, the effect of Sildenafil on the mortality of infants and children with PAH is not clear and further studies are still needed. After an average treatment duration of 4–12 months, the known mortality rate today is still between 5–30%. An insufficient number of studies have been conducted for a clear rate to be determined, but many studies have contributed to a more accurate image of how Sildenafil can be used as a potential therapy, in order to turn the risk and benefit balance in favour of the paediatric patient [51].

## 9. Discussion

The PDE5 inhibitor Sildenafil is a valuable pharmacotherapy that can be used to treat children and infants with PAH, both in monotherapy and combined therapy, as well as for additional related diseases. Nowadays, Sildenafil is approved in Europe for the treatment of PAH in children aged 1–17 years, although with some caution required. The problematic matter of researching new drugs on children is one of the main limitations for the use of Sildenafil, because without proper RCTs being performed, researchers and clinicians cannot be fully aware of the detailed treatment effects or consequent side effects. So far, the use of Sildenafil in the treatment of PAH in infants and children has shown potential benefits and improved patient outcomes. One of the most pressing questions that remains is at what dosage regimen patients can fully benefit from its favourable effects with the minimum possible adverse reactions. As previously mentioned, frequency and severity of adverse effects appear more often at higher doses (20 mg, 40 mg or 80 mg) of Sildenafil and are correlated with higher mortality. At the opposite pole, the administration of low doses (10 mg) were less effective, but have a much lower mortality risk.

Aside from the common aetiology, PAH as a result of cardiovascular surgery and haematological disorders has been alleviated with the use of Sildenafil. Although the haemodynamic and morphological aspects of cardiac transplantation and univentricular heart surgery have been subjected to statistical analyses, in order to assess the efficacy of the PDE5 inhibitor, the data on stem cells transplant remains scarce. The only existing information is from a study with a small group of five children, of whom, the survival of one patient was attributed to the Sildenafil administration. Other indications, such as in PPHN, BPD, CDH and Eisenmenger’s syndrome, have also had positive outcomes of Sildenafil therapy, through the improvement of cardiorespiratory physiology, but the variation in results among paediatric departments renders the need for further evaluation. Additionally, the reports on lymphatic reductions also require further analysis, due to the reduced number of patients in recently conducted studies.

In all cases, it is quintessential that the selection and monitoring of patients has to be thorough, and that every aspect should be evaluated to establish clear criteria of Sildenafil administration, as well as evidence-based data regarding dosage, in order to accomplish the best possible outcome.

## 10. Conclusions

It can be clearly seen that Sildenafil has been proven as a significant potential therapy which can provide beneficial effects in contributing to the improvement of a large variety of conditions. However, the currently available evidence-based data has inconsistent data, inconclusive pharmacological evidence and a lack of exact protocols in different aetiologies of PAH, still create an obstacle for the assessment of the role of Sildenafil, in spite of the recent advances that appear to demonstrate its effectiveness. The potential of Sildenafil alone, and also in combination with other current PAH therapies, requires additional exploration in order to find the correct treatment strategy for the challenging paediatric pathology that is PH. Further research is needed to conclude the ideal posology of Sildenafil and to establish its place in the future developments and therapies in the field of paediatric cardiology.

## Figures and Tables

**Table 1:** Aspects of pulmonary hypertension (PH).

Increased Total Pulmonary Arteriolar Resistance		Increased Pulmonary Blood Flow
Precapillary Site	Postcapillary Site	Congenital Heart Disease
Developmental: No treatment appropriate Pathologic: Requires treatment	Obstruction of the blood flow beyond pulmonary capillary bed Pulmonary vein stenosis Mitral stenosis Left ventricle dysfunction	Atrial septal defect (ASD) Ventricular septal defect (VSD) Persistent ductus arteriosus (PDA) Aorto-pulmonary window Aorto-pulmonary collateral Aneurysm of great vein of Galen

**Table 2:** Most frequent adverse effects to Sildenafil treatment, as seen in the STARTS 1 study.

Number	Adverse Effect	Patients Who Developed Adverse Reactions (*n* = 234)	Percentage
1	Upper respiratory tract infection	53	22.64%
2	Vomiting	45	19.23%
3	Headache	44	18.80%
4	Bronchitis	37	15.81%
5	Pyrexia	36	15.38%
6	Pharyngitis	31	13.24%
7	Cough	30	12.82%
8	Diarrhoea	27	11.53%
9	Nasopharyngitis	25	10.68%
